# Changes in functional connectivity correlate with behavioral gains in stroke patients after therapy using a brain-computer interface device

**DOI:** 10.3389/fneng.2014.00025

**Published:** 2014-07-08

**Authors:** Brittany Mei Young, Zack Nigogosyan, Alexander Remsik, Léo M. Walton, Jie Song, Veena A. Nair, Scott W. Grogan, Mitchell E. Tyler, Dorothy Farrar Edwards, Kristin Caldera, Justin A. Sattin, Justin C. Williams, Vivek Prabhakaran

**Affiliations:** ^1^Department of Radiology, University of Wisconsin – MadisonMadison, WI, USA; ^2^Medical Scientist Training Program, University of Wisconsin – MadisonMadison, WI, USA; ^3^Neuroscience Training Program, University of Wisconsin – MadisonMadison, WI, USA; ^4^Department of Biomedical Engineering, University of Wisconsin – MadisonMadison, WI, USA; ^5^Departments of Kinesiology and Medicine, University of Wisconsin – MadisonMadison, WI, USA; ^6^Department of Orthopedics and Rehabilitation, University of Wisconsin – MadisonMadison, WI, USA; ^7^Department of Neurology, University of Wisconsin – MadisonMadison, WI, USA; ^8^Department of Neurosurgery, University of Wisconsin – MadisonMadison, WI, USA; ^9^Departments of Psychology and Psychiatry, University of Wisconsin – MadisonMadison, WI, USA

**Keywords:** brain-computer interface, stroke rehabilitation, functional connectivity, BCI therapy, UE motor recovery, fMRI

## Abstract

Brain-computer interface (BCI) technology is being incorporated into new stroke rehabilitation devices, but little is known about brain changes associated with its use. We collected anatomical and functional MRI of nine stroke patients with persistent upper extremity motor impairment before, during, and after therapy using a BCI system. Subjects were asked to perform finger tapping of the impaired hand during fMRI. Action Research Arm Test (ARAT), 9-Hole Peg Test (9-HPT), and Stroke Impact Scale (SIS) domains of Hand Function (HF) and Activities of Daily Living (ADL) were also assessed. Group-level analyses examined changes in whole-brain task-based functional connectivity (FC) to seed regions in the motor network observed during and after BCI therapy. Whole-brain FC analyses seeded in each thalamus showed FC increases from baseline at mid-therapy and post-therapy (*p* < 0.05). Changes in FC between seeds at both the network and the connection levels were examined for correlations with changes in behavioral measures. Average motor network FC was increased post-therapy, and changes in average network FC correlated (*p* < 0.05) with changes in performance on ARAT (*R*^2^ = 0.21), 9-HPT (*R*^2^ = 0.41), SIS HF (*R*^2^ = 0.27), and SIS ADL (*R*^2^ = 0.40). Multiple individual connections within the motor network were found to correlate in change from baseline with changes in behavioral measures. Many of these connections involved the thalamus, with change in each of four behavioral measures significantly correlating with change from baseline FC of at least one thalamic connection. These preliminary results show changes in FC that occur with the administration of rehabilitative therapy using a BCI system. The correlations noted between changes in FC measures and changes in behavioral outcomes indicate that both adaptive and maladaptive changes in FC may develop with this therapy and also suggest a brain-behavior relationship that may be stimulated by the neuromodulatory component of BCI therapy.

## Introduction

Decreases in stroke mortality rates began accelerating in the 1970s (Lackland et al., 2014), and reduced stroke mortality was named as one of the 10 great public health achievements in the United States from 2001 to 2010 by the Centers for Disease Control and Prevention (2011). These trends have contributed to a growing population of stroke survivors currently estimated at 4 million individuals in the United States alone (Go et al., 2014). Nevertheless, approximately 795,000 individuals experience a new stroke each year (Go et al., 2014), with up to 50% of survivors suffering from some persistent neurological disability (Kelly-Hayes et al., 2003). Stroke continues to be a leading cause of serious long-term disability, resulting in billions of dollars of economic costs each year (Towfighi and Saver, 2011). Given the magnitude of such costs and the growing population of stroke survivors, there is a need for a better understanding of the mechanisms that underlie stroke recovery and new methods to facilitate stroke rehabilitation.

Functional connectivity (FC) is a measure of the temporal correlation of activation between spatially separate brain regions. Such activation has been shown to be distributed among neuronal areas via functionally or structurally connected networks of neurons (Biswal et al., 1995; James et al., 2009; Nocchi et al., 2012; Jiang et al., 2013; Varkuti et al., 2013). With regard to stroke rehabilitation, the understanding of the FC changes that result from stroke and those observed during the recovery process is a growing area of interest that may be used to guide future therapeutic approaches (James et al., 2009; Grefkes and Fink, 2011; Westlake and Nagarajan, 2011; Jiang et al., 2013; Varkuti et al., 2013). One study of subcortical stroke survivors has shown reorganization in the ipsilesional motor cortex to be strongly associated with post stroke recovery (Zhang et al., 2014). However, another study of subcortical stroke patients found different patterns of increased resting-state FC in the sensorimotor network in those with right hemisphere strokes compared to those with strokes in the left hemisphere (Wang et al., 2014). Clearly, there is much to be learned in the process of characterizing not only changes in FC observed in stroke patients but also in understanding how these changes may be modulated during recovery facilitated by rehabilitative therapies.

Brain-computer interfaces (BCIs) are systems which use detected neural activity to generate real time feedback and present this feedback to the user whose neural activity is being monitored. The user can then use this feedback to learn how to modulate context-specific brain activity. These technologies are being incorporated into a new class of devices intended to facilitate stroke rehabilitation with some success in small-scale studies (Buch et al., 2008; Daly et al., 2009; Broetz et al., 2010; Prasad et al., 2010; Caria et al., 2011; Shindo et al., 2011; Liu et al., 2012; Takahashi et al., 2012). A growing number of studies have shown changes in brain activation associated with imaginary and attempted movements of an impaired upper extremity with the use of these devices in rehabilitative applications intended to improve motor function (Broetz et al., 2010; Caria et al., 2011; Ramos-Murguialday et al., 2013). There is evidence that such changes in activation are accompanied by changes in FC to the areas targeted by training with the BCI system (Rota et al., 2011). In one study of stroke patients, gains in resting-state FC observed after rehabilitative therapy using a BCI device also correlated positively with gains in motor outcomes documented during the same period (Varkuti et al., 2013). However, studies of FC changes observed with the administration BCI therapy remain limited. A more complete understanding of FC changes in response to therapies using BCI systems and how these changes may relate to behavioral outcomes is important in understanding the mechanisms that underlie clinical gains that may be elicited with this new class of devices.

The aim of this paper is to identify changes in motor network FC observed with the administration of interventional rehabilitation therapy using a BCI device and to examine how these changes might relate to behavioral outcomes in stroke patients. We hypothesize that FC in the motor network during finger tapping of the impaired hand will increase with the administration of BCI therapy and that these increases will correlate with gains in behavioral measures assessed outside of the scanner.

## Materials and methods

### Participant recruitment and characteristics

Thirty patients with persistent upper-extremity motor impairment resulting from ischemic or hemorrhagic stroke were contacted regarding study participation. Of these, 16 expressed interest in participating in our study with nine individuals having completed a full course of BCI therapy and MRI assessments thus far. Exclusion criteria included the presence of neurodegenerative disorder (e.g., dementia), other neurological or psychiatric disorders (e.g., schizophrenia), and the inability to provide informed consent. Subjects were also excluded if they had allergies to electrode gel used during the therapy sessions, if they had undergone treatment for recent infectious diseases, if lesions of the oral mucosa were present, if they were pregnant or likely to become pregnant during the course of the study, or if they were unable to safely and comfortably undergo MRI. Of the nine subjects described in this paper (6M, 3F), the average age was 62 years (SD = 9.2 years). Average time from stroke onset was 12.9 months (SD = 7.9 months). More subjects were right-handed (*N* = 7) than left-handed, and more subjects had right-sided impairments (*N* = 7) than left-sided impairments, but these differences were not significant (*p* = 0.18). This study was approved by the University of Wisconsin Health Sciences Institutional Review Board. All subject provided written informed consent prior to participation.

### Intervention schedule and behavioral assessments

Subjects were assessed no more than 1 week prior to the start of BCI therapy (pre-therapy), at the midpoint of therapy, within 1 week after completion of all BCI therapy (post-therapy), and 1 month after the end of the BCI therapy period. Each assessment involved obtaining both behavioral measures as well as MRI scans. Four of the nine subjects were also administered the behavioral assessment at three additional time points during a pre-therapy monitoring period prior to the administration of any BCI therapy. Behavioral measures administered at each assessment included the Action Research Arm Test (Carroll, 1965; Lang et al., 2006), the 9-Hole Peg Test (9-HPT) (Beebe and Lang, 2009), and the Stroke Impact Scale (SIS; Duncan et al., 1999; Carod-Artal et al., 2008). Scores for the 9-HPT were calculated as the average of two attempts using the impaired hand. ARAT scores reflect a total score assigned for the subject’s impaired hand. Of the domains of the SIS, this study focused on the Activities of Daily Living (SIS ADL) and Hand Function (SIS HF) domains, as these represent the domain most closely related to the motor functions practiced with the BCI therapy administered (SIS HF) and the domain most reflective of global function (SIS ADL) that may inform the clinical implications of the results. SIS domain scores were transformed to yield a percentage of possible points obtained, in accordance with standard SIS scoring practice. All SIS domain scores discussed in this paper refer to these transformed scores.

### BCI therapy and session sequence

All subjects received up to 15 2-h sessions of interventional therapy using an EEG-guided BCI device, which incorporated visual display, tongue stimulation, and functional electrical stimulation as feedback. These BCI therapy sessions were scheduled over the course of up to 6 weeks with no more than three sessions per week.

All BCI therapy was set up using BCI2000 software (Schalk et al., 2004) version 2 with in-house modifications to allow for administration of additional tongue stimulation (TDU 01.30, Wicab Inc.) and functional electrical stimulation (LG-7500, LGMedSupply; Arduino 1.0.4). A 16-channel EEG cap and amplifier (Guger Technologies) were used for the detection and recording of all EEG signals during the BCI therapy sessions. Each 2-h session began first with an open-loop screening task used to identify appropriate control signals. During the screening task, the subject was cued to perform attempted movement of either hand alternating with periods of rest using the cues “right”, “left”, and “rest”. The specific movements used varied across subjects, as the specific movements used were individualized to the baseline abilities and recovery goals of each patient. Repeated opening and closing of the hand and wrist extension were common choices among subjects in this study, although some chose to use wrist flexion or squeezing motions. Cues were shown as words on a screen one at a time in blocks of 4 s. Each cue was shown at least 10 times at the beginning of each session. During this screening task, no feedback was provided to the subject. Data collected during these open-loop trials then determined appropriate EEG-based control features to guide all subsequent closed-loop tasks, using a process previously described to determine optimal control features (Wilson et al., 2009).

Attempted movement rather than motor imagery was used for both initial screening and for subsequent closed-loop feedback conditions with the intent of making the training conditions of the neurofeedback task as similar as possible to the mental processes invoked when attempting functional real world movement. This was done because when using the BCI system as an assistive feedback device for rehabilitative therapy to help reestablish function, rather than as an augmentative means of replacing a lost function, it is important for the effects of the training to be accessible to the subject beyond the laboratory environment. Therefore, we based control signals on neural activity patterns generated during attempted movements in an attempt to maximize the extent to which the context-specific strengthening of movement-related patterns of brain activity might persist and benefit the individual when attempting movements beyond the therapy period.

While many BCI systems were originally controlled using motor imagery alone, these early systems were developed using individuals without motor impairments with motor imagery used as a way to establish the ability to control a BCI device independent of the production of normal movements (Leuthardt et al., 2004). Later studies demonstrating the ability of impaired individuals to successfully achieve similar control of BCI devices as seen with healthy subjects continued with the use of motor imagery, recreating similar task conditions to those with which healthy subjects were trained and often relying on paradigms that did not target use of actual deficits present in the impaired populations tested (Wolpaw and Mcfarland, 2004). It may also be important to acknowledge that many early BCI devices were developed to function as augmentative devices rather than for rehabilitative purposes, which may partially explain the heavy emphasis on using mental tasks like motor imagery that could be performed consistently in the absence of any actual movement over mental tasks that might also produce more heterogeneous amounts of physical movements due to variations in the severity of deficits among subjects. Therefore, while these early systems established a precedent of motor imagery as a standard method for training with motor-oriented BCI devices, this tradition does not preclude the ability of an individual to control the device with mental tasks other than motor imagery and even with brain regions anatomically distinct from the sensorimotor cortical areas that are often used (Felton et al., 2007).

BCI devices that rely on motor imagery continue to be used today for both rehabilitative (Buch et al., 2008; Daly et al., 2009; Broetz et al., 2010; Prasad et al., 2010; Caria et al., 2011; Shindo et al., 2011; Varkuti et al., 2013) and augmentative (Kubler et al., 2005) purposes, although newer systems have also incorporated actual movement into their protocols using BCI systems for rehabilitative purposes with some success (Daly et al., 2009; Prasad et al., 2010; Takahashi et al., 2012; Ramos-Murguialday et al., 2013; Mukaino et al., 2014). While therapy using our BCI system encourages actual attempted movements rather than imagined movements during both open-loop and closed-loop conditions, we believe that this system can still be classified as a BCI as the feedback provided by the device is controlled purely by neural signals detected by EEG, creating a real time interface between the brain and the computer-generated stimuli.

Subjects were then taught to perform a closed-loop task during which real time visual feedback was presented to help the subject learn to modulate cortical activity during attempted movement of each hand. This feedback was presented in the context of a game. The subject was instructed to move a cursor onto a target area. Target areas were presented on either the left or right side of the screen, and subjects were instructed to use movement of the left or right hand to move the cursor in the left or right direction respectively. Lateral cursor movement was determined by the subject’s real time EEG signals, with cortical activity associated with attempted left (right) hand movement translating to leftward (rightward) movement of the cursor. At each therapy session, subjects first completed a goal of at least 10 runs, each run consisting of 8–12 trials and each trial presenting one of four targets, with visual feedback alone as described.

After approximately 10 trials with visual feedback alone, functional electrical stimulation and tongue stimulation were added to the same game play task. Functional electrical stimulation was applied to the muscles of the impaired arm and triggered such that electrical stimulus was only delivered when appropriate neural activity signals corresponding to attempted movement of the impaired hand were detected on EEG during a trial in which it was necessary to move the cursor toward a target on the impaired side of the body. Tongue stimulation paralleled the spatial information of visual feedback, providing continuous electrotactile stimulation of the tongue on an electrode grid during each trial. Stimulus delivered in areas of the tongue stimulation grid represented the positions of the cursor and target on screen.

Subjects were allowed to take short breaks between trials if desired or upon request. In order to keep the sessions more interesting, game dynamics (e.g., size of the target) could be changed to make the task more difficult once subjects achieved adequate cursor control and accuracy (approximately 70% of targets attained) at a given level of difficulty. Similarly, if a subject who had previously been advanced to a more difficult level of game play showed a sudden reduction in their ability to control the cursor, the level of difficulty could be reduced temporarily as the subject reattained sufficient control to increase the proportion of targets attained. These adjustments were made to help keep the subject engaged at a task that is consistently challenging enough to minimize boredom but not so challenging that the subject loses motivation.

### MRI task instructions

Subjects were scanned during a block-design task fMRI during which they were instructed to alternate finger tapping of the impaired hand with rest in 20 s blocks. Cues to the tap and rest conditions were given using either visual or tactile cues. Subjects unable to generate tapping movements on their own performed assisted tapping during tap blocks. For both functional and anatomical scans, subjects were instructed to lie still and attempt to minimize any head movements.

### MRI acquisition and processing

All MR images were acquired on one of two GE MR750 3T scanners (GE, Milwaukee, Wisconsin) equipped with high-speed gradients and using an 8-channel head coil. Padding around subjects’ heads was used to help minimize movement. Functional scans were obtained using a T2*-weighted gradient-echo echo planar imaging (EPI) pulse sequence sensitive to BOLD contrast: field of view 224 mm, matrix 64 × 64, TR 2600 ms, TE 22 ms, flip angle 60°, acquiring 40 axial plane slices of 3.5 mm thickness with 3.5 mm spacing between slices. In total, 70 sequential whole-brain acquisitions were recorded during each functional scan. A T1-weighted high-resolution anatomical image was also obtained during each scanning session using a BRAVO FSPGR pulse sequence: field of view 256 mm, matrix 256 × 256, TR 8.16 ms, TE 3.18 ms, flip angle 12°, and 156 axial plane slices of 1 mm thickness with 1 mm spacing between slices.

All processing and pre-processing of MR scans was performed using Analysis of Functional NeuroImages (AFNI; Cox, 1996). The first four volumes of each functional scan were removed to allow for signal stabilization. Functional data sets were motion corrected and spatially smoothed at 6 mm with a full width at half maximum Gaussian kernel. Each voxel time-series was scaled to a mean of 100, and six motion parameters were regressed out. EPI data sets were visually inspected for alignment with anatomical T1 datasets, using align_epi_anat.py to align the anatomical T1 scan to the EPI data set if alignment was not acceptable upon first inspection.

Each of seven regions previously identified as components of the motor network (Shirer et al., 2012) was seeded with an ROI of radius 6 mm. Regions were the left thalamus, right thalamus, left primary motor cortex, right primary motor cortex, right supplementary motor area, left cerebellum, and right cerebellum. Whole-brain connectivity analyses to each seed region were conducted using the motion regressed residual time-series derived from the functional scans. Whole-brain connectivity maps were then transformed into a standardized brain space (Talairach and Tournoux, 1988). For the two subjects with right-sided stroke lesions, images were flipped along the mid-sagittal line so that in group-level comparisons the left hemisphere was ipsilesional and the right hemisphere was contralesional.

### Statistical analysis

All statistical analyses were conducted using either AFNI (Cox, 1996) software or in *R* statistical software (version 3.0.1). A paired *t*-test was used to identify areas of significant connectivity change at the group level. Maps were cluster corrected for multiple comparisons, with minimal cluster size 300 voxels, and thresholded at *t* ≥ 2.366 (*p* < 0.05). Within-subject correlation matrices for the 21 (7 × (7 − 1)/2) pairs of ROI seeds were computed from motion regressed residual time-series for each subject. The Fisher *Z*-transform was applied to each of these correlation coefficients to produce measures of an approximately normal distribution, and these transformed coefficients were then used in all subsequent analyses.

Average network connectivity was calculated by averaging all 21 transformed correlation coefficients representing functional connections between each pair of seeds in the motor network. Connections were classified as either interhemispheric or intrahemispheric, and these groups were analyzed for differences using linear mixed effects modeling.

Changes from pre-therapy baseline in correlation strength between each pair of seed regions were analyzed for correlation with changes from baseline behavioral measures. This was done first by calculating Pearson’s *r* and applying fdr correction to the resulting *p*-values obtained for each correlation examined, making the assumption of independence among data points. Recognizing that these data follow the same individuals at multiple time points and are therefore not truly independent, a follow-up analysis of correlations found to be significant using Pearson’s *r* and fdr correction was conducted using generalized estimating equations (GEE), which does account for the repeated measures component of the data collected but requires the assumption that an independent and dependent variable can be named in the model. Subjects that exhibited floor or ceiling effects in behavioral measures were excluded from correlation analyses of that measure.

## Results

### Behavioral measures

Group performance on each of the four behavioral measures assessed is summarized in Table 1. No significant group differences in behavioral measures were found when comparing mid-therapy, post-therapy, and 1 month post therapy assessment scores with pre-therapy baseline measures in any of the four behavioral outcomes. The average amount of change observed in behavioral measures during the pre-therapy period and during the BCI therapy period for each of the four subjects who completed three additional behavioral assessments prior to the start of BCI therapy is presented in Table 2. Changes for these subjects during the pre-therapy period were smaller in magnitude for the ARAT and SIS HF scores. Average change in SIS ADL was negative for all four subjects during the waitlist period, with positive changes noted during the BCI therapy period in three of the four.

**Table 1:** **Group averages on each of four behavioral measures at each of four time points**.

	**ARAT (points)**	**9-HPT (seconds)**	**SIS ADL (score)**	**SIS HF (score)**
**Pre-therapy**	27.78 (9.06)	52.59 (7.89)	68.06 (6.30)	26.11 (9.67)
**Mid-therapy**	26.44 (8.60)	52.10 (7.53)	72.22 (6.28)	30.56 (10.59)
**Post-therapy**	28.11 (9.03)	53.13 (6.70)	68.89 (5.59)	28.89 (10.30)
**One month after BCI therapy**	25.38 (10.09)	41.33 (7.00)	70.31 (6.02)	25.31 (10.74)

*Numbers shown are group averages with standard error of the mean in parentheses. Scores for ARAT, SIS ADL, and SIS HF reflect data from all nine subjects. Scores for 9-HPT reflect values of only the four subjects who were able to complete the task within the allotted 5 min. ARAT = Action Research Arm Test, 9-HPT = Nine-Hole Peg Test, SIS ADL = Stroke Impact Scale—Activities of Daily Living, SIS HF = Stroke Impact Scale—Hand Function*.

**Table 2:** **Average change observed during pre-therapy and BCI therapy periods for each of four subjects with additional behavioral assessments prior to the start of BCI therapy**.

	**ARAT (points)**		**9-HPT (seconds)**		**SIS-ADL (score)**		**SIS-HF (score)**	
**Subject**	**Pre-Therapy**	**BCI Therapy**	**Pre-Therapy**	**BCI Therapy**	**Pre-Therapy**	**BCI Therapy**	**Pre-Therapy**	**BCI Therapy**
**A**	Floor	Floor	Unable	Unable	−11.67	5	1.67	−5
**B**	−0.33	2.5	−33.51	−3.49	−2.5	3.75	−1.67	0
**C**	2.67	10	Unable	Unable	−11.67	2.5	0	26.67
**D**	2	−4.33	−26.11	17.52	−2.5	−21.67	18.33	−21.67

*Floor = a floor effect was observed in which the subject was unable to score points for the measure at any time point assessed. Unable = subject was unable to perform the task within the allotted 5 min time period. ARAT = Action Research Arm Test, 9-HPT = Nine-Hole Peg Test, SIS ADL = Stroke Impact Scale—Activities of Daily Living, SIS HF = Stroke Impact Scale—Hand Function*.

### BCI performance results

**Figure 1:**
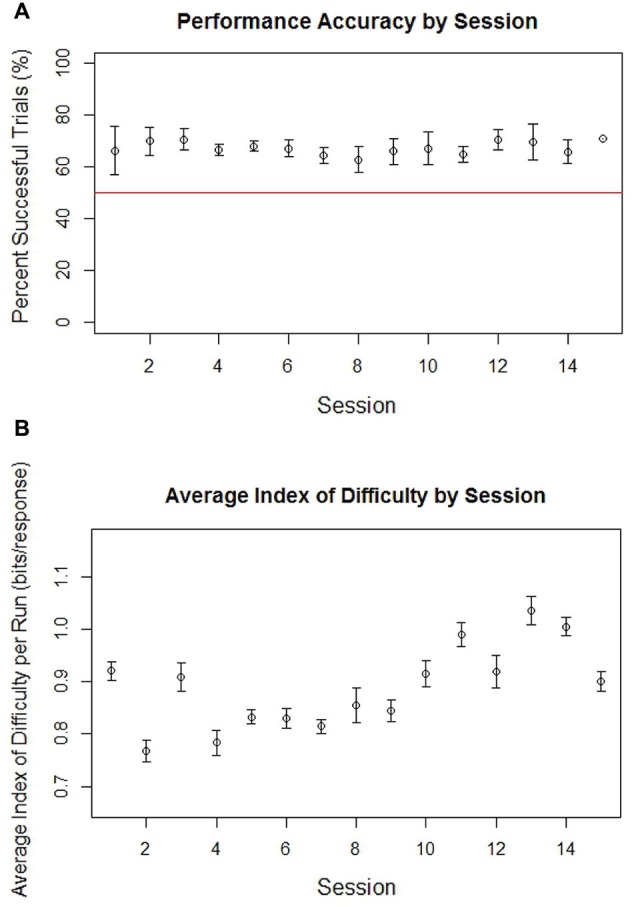
**Average accuracy in percent successful trials (A) and average Fitt’s Index of Difficulty (B) by run for all non-adaptive runs of BCI cursor task game play across all subjects grouped by session**. The red line in the graph of accuracy for each session is drawn at 0.5, which represents the level at which half of trials would be successful. Error bars represent the standard error.

The average performance accuracy across all subjects for the BCI cursor task for each session is shown in Figure 1A. Subjects were able to consistently maintain an average accuracy above 0.5, meaning that on average subjects were able to successfully control the cursor well enough to achieve successful target attainment in over half the trials. This maintenance of greater than 50% overall accuracy throughout the therapy period was accompanied by a general increase in the average Fitts’s Index of Difficulty (Fitts, 1954) for each session, as can be seen in Figure 1B. The pattern of relatively constant accuracy over sessions along with a general increase in difficulty is consistent with the method of increasing task difficulty at an individualized pace, advancing individual subjects to more difficult game play parameters once they able to increase accuracy on individual runs to approximately 70%. Individually, a binomial test of each subject’s performance showed individual accuracies across all completed non-adaptive runs to be significantly greater than chance (*p* < 0.05 for each subject). Due to a computer error, BCI performance data was lost for a small number of runs; however, these lost runs represented less than 4% of all applicable data and were not known to be different from the remaining 96% of runs used in the analysis presented.

### fMRI whole-brain connectivity analysis

**Figure 2:**
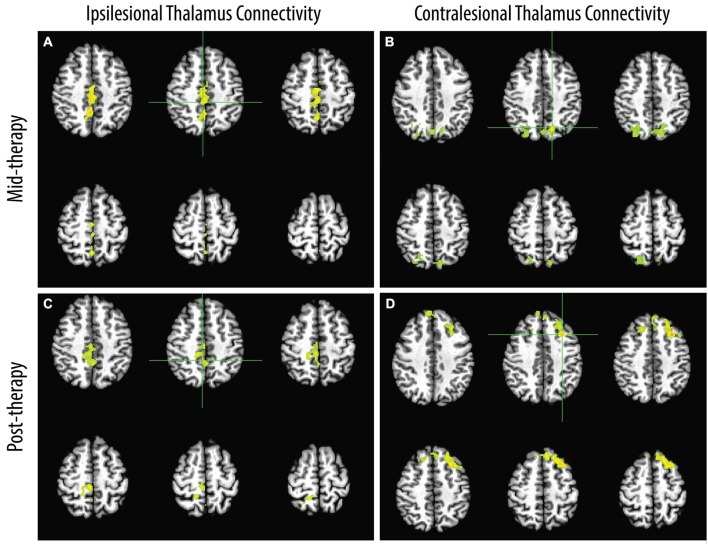
**Areas of group-level increases from pre-therapy baseline in FC to seeds in the ipsilesional (A, C) and contralesional (B, D) thalami observed mid-therapy and upon completion of therapy**. Hemispheres shown on the right side in the image correspond to the ipsilesional hemisphere.

Maps of significant increases in FC to thalamic seed regions are shown in Figure 2. At mid-therapy, increases in FC were noted between the ipsilesional thalamus seed and parts of the bilateral precuneus and bilateral cingulate. Increases were also noted between the contralesional thalamus and the contralesional cerebellum as well as with the bilateral precuneus. Upon completion of BCI therapy, increases were noted between the ipsilesional thalamus and the contralesional cingulate, contralateral paracentral lobule, and the bilateral precuneus. Increases were also noted between the contralesional thalamus and the bilateral anterior cingulate and the ipsilesional superior and middle frontal gyri. Significant group-level changes in FC at mid-therapy and as well as upon completion of therapy were not observed for other seed regions.

### Network connectivity analysis

The average connection strength over the entire network was increased from baseline upon completion of BCI therapy, but this increase was not statistically significant. No individual connections within the network showed significant changes from baseline at the group level during the study period.

**Figure 3:**
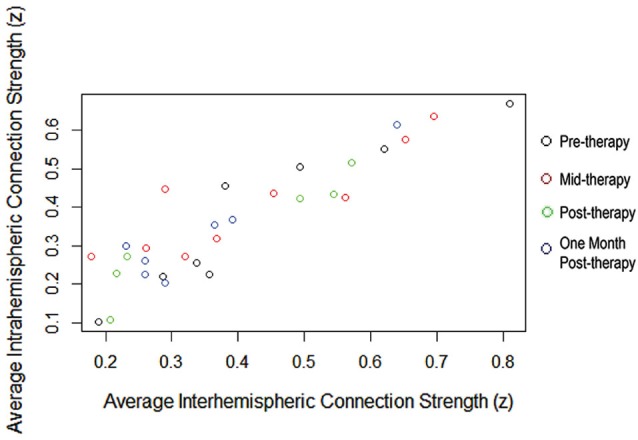
**Average within-subject interhemispheric and intrahemispheric connection strengths correlated throughout the BCI therapy study period**.

Analyses of the interhemispheric and intrahemispheric components of the network showed no significant differences between the two types of connections in connection strength or in patterns of change over the study period. As shown in Figure 3, the average within-subject interhemispheric connection strength correlated with average within-subject intrahemispheric connection strength using a Pearson’s *r* calculation (*R*^2^ range 0.77–0.95; *p* < 0.002 at all four time points).

### Behavioral correlations with changes in motor network connectivity

**Figure 4:**
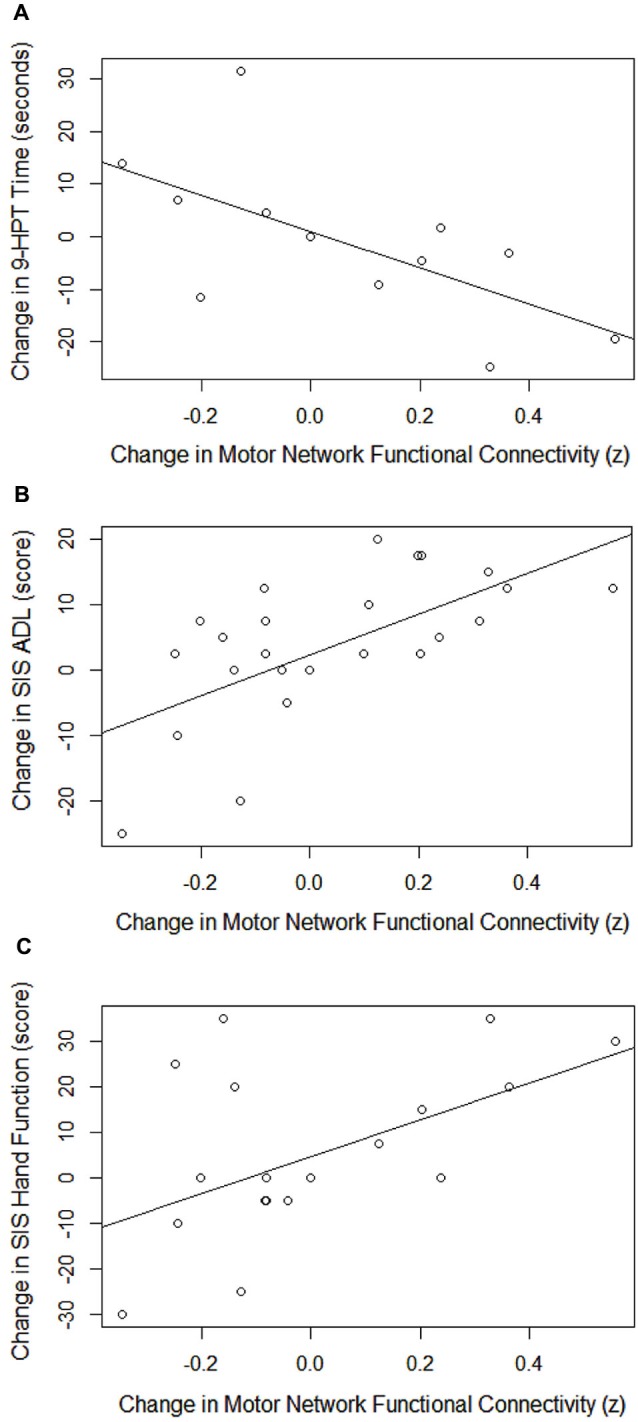
**Correlations between changes in motor network FC with changes in 9-HPT performance (A), SIS ADL scores (B), and SIS HF scores (C)**. 9-HPT = Nine-Hole Peg Test, SIS ADL = Stroke Impact Scale—Activities of Daily Living, SIS HF = Stroke Impact Scale—Hand Function.

Changes from pre-therapy baseline in average within-subject network connectivity across the pre-, mid-, post-, and 1 month after- therapy time points correlated to changes in all four behavioral measures using Pearson’s *r*. As shown in Table 3, these relationships remained significant using a GEE approach for the 9-HPT (Figure 4A), SIS ADL (Figure 4B), and SIS HF (Figure 4C) measures.

**Table 3:** **Correlations between changes in motor network connectivity with changes in behavioral outcome measures**.

**Measure**	**Pearson’s *r***	**Correlation *p*-value**	**GEE *p*-value**
**ARAT**	−0.458	0.049*	0.345
**9-HPT**	−0.640	0.010*	3.303 × 10^−6^*
**SIS ADL**	0.635	1.023 × 10^−4^*	1.796 × 10^−4^*
**SIS HF**	0.518	0.011*	0.038*

*Significant at p < 0.05. ARAT = Action Research Arm Test, 9-HPT = Nine-Hole Peg Test, SIS ADL = Stroke Impact Scale—Activities of Daily Living, SIS HF = Stroke Impact Scale—Hand Function.

### Behavioral correlations with changes in individual connection strengths

A summary of Pearson’s correlations found to be significant after fdr correction that also survived GEE analysis between changes in individual connection strength within the motor network and changes in behavioral scores is presented in Table 4. At least one connection achieved significance using both methods for each of the four behavioral measures examined. Of these connections, the majority were connections involving either the ipsilesional or contralesional thalamus.

**Table 4:** **Correlations between changes in connection strength of individual motor network functional connections and changes in behavioral outcome measures**.

Behavioral Measure	Seed-seed Covariation	Pearson’s *r*	Correlation fdr-corrected *p*-value	GEE analysis *p*-value
**9-HPT**	I thalamus—C primary motor cortex	−0.669	0.023	0.009
	I thalamus—I primary motor cortex	−0.678	0.023	8.123 × 10^−75^
	C thalamus—C primary motor cortex	−0.851	6.36 × 10^−4^	1.110 × 10^−6^
**ARAT**	I thalamus—I cerebellum	−0.677	0.011	0.027
	C primary motor cortex—C supplementary motor area	−0.677	0.011	7.213 × 10^−5^
**SIS HF**	I thalamus—C cerebellum	0.790	8.028 × 10^−5^	0.006
	I cerebellum—C cerebellum	0.547	0.025	0.016
	I thalamus—I primary motor cortex	0.632	0.007	0.003
**SIS ADL**	C thalamus—C primary motor cortex	0.686	2.315 × 10^−4^	0.001

*I = ipsilesional, C = contralesional, ARAT = Action Research Arm Test, 9-HPT = Nine-Hole Peg Test, SIS ADL = Stroke Impact Scale—Activities of Daily Living, SIS HF = Stroke Impact Scale—Hand Function*.

## Discussion

The results of these preliminary analyses are suggestive of a relationship between the changes in FC and those in behavioral outcomes observed with the administration of BCI therapy. Although no significant behavioral gains were demonstrated at the group level, behavioral measure changes during the pre-therapy and BCI therapy periods for subjects assessed at additional time points prior to intervention suggest that on the individual level there may still be a differential response to therapy using the BCI system not explained by practice effects on different behavioral tasks. The lack of significant group changes over the course of the therapy period in overall FC at the network and connection levels may be due to the small number of individuals included in these analyses, rendering them underpowered to identify more subtle changes that may have been present.

The thalamus is a component of the motor network (Shirer et al., 2012) that lesion studies in both primates (Bornschlegl and Asanuma, 1987; Canavan et al., 1989) and humans (Lee and Marsden, 1994) have shown to be important for normal motor learning and motor function. In the context of motor recovery after stroke, increases in ipsilesional thalamic activation have been shown to correlate with motor performance in chronic stroke patients undergoing treadmill training (Enzinger et al., 2009), and abnormalities in resting-state FC with the thalamus have been found in stroke patients, with some of these connections found to correlate with motor outcomes (Wang et al., 2010; Park et al., 2011). One longitudinal study of subcortical stroke patients observed changes in FC between the ipsilesional thalamus and contralesional areas that correlated with functional outcomes during the first year following stroke (Wang et al., 2010). Another study focusing on supratentorial stroke patients found increased FC between the ipsilesional motor cortex and the bilateral thalamus compared to healthy controls, with FC between the ipsilesional motor cortex and the contralesional thalamus correlating positively with motor recovery 6 months following stroke (Park et al., 2011).

While the use of an EEG-guided BCI device rewards the modulation of largely cortical neural activity without direct feedback intended to target subcortical activity due to the nature of EEG, the finding of significant changes in FC to each thalamus in these patients indicates that changes in thalamic FC may be encouraged by the brain-driven nature of BCI therapy. These increases may relate to the fact that feedback from the BCI device used in this study is controlled in part by desynchronization of the mu rhythm over the sensorimotor cortex, which is thought to be produced by thalamocortical circuits (Niedermeyer and da Silva, 2005). Furthermore, it is possible that the achievement-based constraints of the BCI-guided task employed in this therapy, which encourages and rewards appropriate cortical activity with the attainment of a target that can be counted or scored, contribute to this effect. The areas observed to have increased FC to the ipsilesional and contralesional thalami in this study, in particular the precuneus, cerebellum, cingulate, and anterior cingulate, have been implicated in a number of cognitive processes including visuospatial imagery (Cavanna and Trimble, 2006; Timmann and Daum, 2007), motor coordination and learning (Fine et al., 2002), attention (Timmann and Daum, 2007), learning (Bussey et al., 1996), memory (Kozlovskiy et al., 2012), and reward-based learning (Shenhav et al., 2013). These results suggest that a learning process may constitute a critical component of therapy using this BCI device.

Subject performance on the BCI task, which was maintained a relatively constant accuracy over sessions while task difficulty gradually increased with time (Figure 1), also supports the hypothesis that therapy using this BCI system promotes an adaptive learning process. In this experiment, subjects effectively learned to achieve greater and greater degrees of neuromodulatory control in order to regain a target level of accuracy when faced with increasingly difficult tasks. In contrast to the phenomena of learned non-use sometimes observed in stroke survivors (Taub, 1976), BCI therapy allows for the practice of modulating behavioral, muscular, and neuronal output for goal attainment in a novel learning environment with multi-modal feedback. If viewed as an operant conditioning mechanism, BCI therapy may thereby facilitate neuroplasticity through this reinforcement of central-peripheral connections (Hebb, 2005).

That the connections found to correlate in degree of change with changes in behavioral outcomes were also largely thalamic connections further underscores the importance of understanding the role of thalamic FC in the process of motor recovery after stroke. An examination of the relationships between these individual connections—thalamic and non-thalamic—and the behavioral measures studied also shows that both adaptive and maladaptive changes may have been present. Namely, while performance on most behavioral measures studied tended to improve with improved FC strength, ARAT performance was negatively correlated with increased connectivity between the ipsilesional thalamus and ipsilesional cerebellum and between the contralesional primary motor cortex and the contralesional supplemental motor area. The simultaneous development of adaptive and maladaptive changes in functional connection strengths during the process of stroke recovery has been documented in a previous study (Wang et al., 2010) similar to the findings presented here, while others have identified changes in FC that may facilitate motor recovery (Rosso et al., 2013). The role of such changes in the process of stroke recovery and the way in which such changes may be modulated through the use of traditional and experimental rehabilitative therapies remains to be fully characterized and understood. However, the presence of both adaptive and maladaptive connections may represent a period in an ongoing recovery process in which both types of connections are formed and subsequently modified, similar to that of synaptic pruning and strengthening observed during the learning and aging processes of normal development (Hebb, 2005).

Another factor that may have contributed to the development of maladaptive functional connections is the possibility that insufficient or unreliable feedback from the BCI device, which might be evidenced by poor BCI task performance, could result in frustrating the participant with the unintentional reinforcement of maladaptive neural activity patterns. While the subjects in this study were able to perform significantly better than chance when using the BCI device, performance accuracy was not necessarily at or above 70%—a common threshold used to indicate adequate BCI control. However, the use of 70% as a threshold for BCI performance appears to stem from earlier studies in which this level of accuracy was needed for communication using a Language Support Program (Kubler et al., 2001, 2005). There appears to be less evidence supporting the use of 70% as a threshold for performance accuracy when no communicative purpose is intended for the BCI system, and in fact significant variation in individual ability to control similar BCI cursor tasks can persist even after 10 sessions of training (McFarland et al., 2005).

It is also known that at least some of the literature exploring human performance in BCI tasks demonstrating consistent performance greater than 70% tends to be biased in favor of good performers. This bias stems from the fact that poor performers are seldom invited to return for further testing (Fazli et al., 2009), do not always have their performance accuracy reported (McFarland et al., 2008), and are not always allowed to continue the full course of BCI training, thereby excluding them from final accuracy reports (Wolpaw et al., 1991). In studies with no indication that good performers have been preferentially enrolled, accuracies over approximately 20 or more training sessions on BCI cursor tasks can range from just above 20% to >90% depending on game play parameters and individual performance (Wolpaw and Mcfarland, 1994, 2004; McFarland and Wolpaw, 2003). Furthermore, game play parameters were dynamically adjusted in this study to make the game more difficult as subjects achieved 70% accuracy at a given level or difficulty. Therefore, subject performance in this study is not necessarily expected to be at or above 70% when averaged over all trials because subjects were not given the opportunity to perform with greater than 70–80% accuracy for long stretches of training without game difficulty increasing.

While subject performance accuracy is readily calculable based on the numbers of successful and unsuccessful trials, it is more difficult to assess the accuracy of the feedback presented based on task performance. This is because a subject may fail to achieve greater than 70% accuracy even with a perfectly calibrated BCI feedback system if the individual’s ability to modulate their Mu/Beta desynchronization is not well controlled. Still, it may be that imperfect feedback effectively lowered the performance accuracy of the subjects studied, thereby frustrating the subjects and affecting subject motivation and engagement with the therapy task. Imperfect feedback may also have contributed to the formation of the maladaptive changes in FC observed, potentially rewarding suboptimal patterns of neural activity during movement attempts or discouraging patterns that would have been truly optimal. Nevertheless, it is worth noting that even imperfect feedback during BCI therapy would constitute a more reliable form of neurofeedback than the absence of such feedback that is available during standard of care therapies such as traditional physical and occupational therapy.

It will be key in future studies to determine whether the rehabilitative effects of BCI therapy can be adequately achieved with the attempted use of an imperfect feedback system or if both feedback accuracy and subject performance accuracy should be further optimized before additional feedback modalities and increased task difficulty are applied. In conducting the experiment described in this paper, many subjects actually expressed a desire for the task to be made more difficult so that the game was not “too easy” or “boring”, even in cases where the participants were not consistently achieving 80–100% accuracy. Changes in game parameters to increase task difficulty appeared to help keep subjects engaged with the BCI therapy. To this end, future studies may also serve to better characterize the tradeoff between increased thresholds for feedback and performance accuracy and the benefits of maintaining subject engagement and motivation throughout the therapy period.

There has been much interest in understanding the relative contributions of interhemispheric and intrahemispheric FC to motor recovery after stroke. Studies of acute stroke patients have shown decreases in interhemispheric connectivity in the motor cortex compared to healthy controls (Carter et al., 2010; Golestani et al., 2013; Xu et al., 2014). Such disruptions have been shown to correlate with ARAT performance in the acute stage of stroke only when interhemispheric connections are considered (Carter et al., 2010). Similarly, increases in interhemispheric but not intrahemispheric FC between homologous regions of the primary sensorimotor cortex have been shown to correlate with Motricity Index scores during the first year of recovery (Xu et al., 2014), and some patients with full recovery 90 days after stroke show a return to normal motor connectivity within the same time frame (Golestani et al., 2013). Homotopic interhemispheric connectivity of the motor cortex has also been shown to correlate with motor function in chronic stroke patients (Chen and Schlaug, 2013; Urbin et al., 2014). Our analysis found no such differentiation between interhemispheric and intrahemispheric functional connections, suggesting that the BCI therapy administered contributed to brain-behavior changes mediated by a whole-network effect, with both individual intrahemispheric and interhemispheric connections showing significant relationships with behavioral gains. Nevertheless, it may be worth noting that both individual functional connections found to be negatively correlated with changes in ARAT performance were intrahemispheric. Few studies have explored changes in FC with BCI therapy. In one study, increases in FC between the bilateral supplemental motor area and areas beyond the motor network correlated positively with gains in Fugl-Meyer scores after BCI therapy (Varkuti et al., 2013). Future studies of FC changes after stroke with spontaneous and facilitated recovery will enhance the understanding of how these changes relate to motor gains and how such relationships may differ based on patient and therapy characteristics.

At the network level, it has been suggested that motor recovery following stroke may be enhanced with reactivation of the motor network along with therapy that allows for adaptive motor network reorganization (Calautti and Baron, 2003). The strong correlation between the average strengths of inter- and intrahemispheric connections throughout the therapy period also supports the idea that the changes in FC seen with BCI therapy in this group of subjects were effected at the whole-network level rather than through the selective strengthening or weakening of one class of connections. Increases in connectivity within the motor network also correlated to changes in 9-HPT performance as well as changes in SIS ADL and SIS HF scores. It is important to note that this relationship with 9-HPT performance can only be said to exist among the higher-functioning subjects included in the study, as those with more significant impairments were unable to complete the assessment and therefore omitted from the analysis. While different patterns of FC change have been documented in severely impaired stroke patients relative to their mildly impaired counterparts (Rosso et al., 2013), the persistence of significant relationships between motor network connectivity and 9-HPT performance as well as with the subjective SIS domain scores, which included subjects at all levels of impairment, suggests the presence of a similar effect in the more impaired subjects as well. Furthermore, the difference in behavioral measure changes during the pre-therapy period relative to those observed during the BCI therapy period hints that the BCI therapy administered may be implicated in the effect.

Unfortunately it is not possible at this time to make statistical comparisons with the limited amount of data from subjects who underwent additional assessments during a pre-therapy period, which would allow for a clearer understanding of the effect of the BCI therapy administered. It is similarly difficult to disambiguate the relative contributions of the various components of therapy using this BCI system to the effects observed. This study remains limited in that it cannot establish the degree to which the changes observed are attributable to the neurofeedback aspect of the BCI therapy and how much of these same phenomena may be due to other aspects of therapy with this system, such as repetitive practice or functional electrical stimulation. Nevertheless, BCI performance results suggest that participant engagement with the neurofeedback task persisted throughout the therapy period, and both BCI performance and neuroimaging findings support the existence of an active learning process over the course of therapy as previously discussed. Therefore, it would seem that the neurofeedback component of BCI therapy is likely to contribute to the effects described in this study, as it demands consistent engagement from each participant and is the only component of the BCI system described that allows for reward-based neuromodulatory training.

Other limitations of this work include the use of a relatively small sample of heterogeneous subjects, which may have resulted in some analyses being insufficiently powered to detect smaller changes or more subtle relationships, especially after floor and ceiling effects were accounted for in analyses that incorporated behavioral data. The brain-behavior relationships observed also remain correlational, with potential causal mechanisms underlying these relationships only speculative based on the data available. To help address some of these issues, future work will aim to study larger cohorts with a more robust seeding of the motor network in order to allow for a more thorough representation of motor network regions and a better description of the FC changes that occur. Future studies will also benefit from the incorporation of data from a larger pre-therapy or control group in order to differentiate the effects of the BCI therapy from other factors such as practice effects, as well as from designs in which groups receiving passive repetitive stimuli can be compared to groups receiving stimuli triggered by neurofeedback in order to better isolate the effect of the neurofeedback component of these types of devices. As the understanding of the mechanisms by which BCI therapies may induce changes in both FC and behavioral motor function improves, these insights may be used to guide the development of future devices better targeted to the needs of the patients who use them.

## Author contributions

Brittany M. Young assisted in subject recruitment, data collection, data analysis, and writing. Zack Nigogosyan assisted with data collection and writing. Alexander Remsik assisted with data collection and writing. Léo M. Walton assisted with data collection and writing. Jie Song assisted with subject recruitment and data collection. Veena A. Nair assisted with subject recruitment, data collection, data analysis, and writing. Scott W. Grogan assisted in data collection. Mitchell E. Tyler provided TDU hardware and expertise. Dorothy F. Edwards assisted with study design and data analysis. Kristin Caldera assisted with subject recruitment. Justin A. Sattin assisted with study design and subject recruitment. Justin C. Williams is one of two lead PI’s on this project and supervised the technical and engineering aspects of the work. Vivek Prabhakaran is one of two lead PI’s on this project and supervised the neuroimaging and neuroscience aspects of this work.

## Conflict of interest statement

There is one patent pending on the closed-loop neurofeedback device used for the therapy administered in this study (Pending U.S. Patent Application No. 12/715,090). This patent was filed jointly by the two lead investigators Justin C. Williams and Vivek Prabhakaran. Otherwise, the authors have no conflicts of interest to report, as this research was conducted in the absence of commercial and financial relationships that might compromise the integrity of the results reported herein.
